# Robotic-assisted lymphovenous anastomosis to treat periorbital lymphedema and systematic review of lymphatic reconstruction of face and neck lymphedema

**DOI:** 10.1007/s11701-025-02552-6

**Published:** 2025-07-12

**Authors:** Carlotta Imholz, Claudine Schaller, Jennifer A. Watson, Carmen E. Zurfluh, Alexandru Grigorean, Nicole Lindenblatt

**Affiliations:** 1 Department of Plastic and Hand Surgery, University Hospital Zurich, Rämistrasse 100, 8091 Zurich, Switzerland; 2 Department of Angiology, University Hospital Zurich, Zurich, Switzerland; 3 University of Zurich, Zurich, Switzerland

**Keywords:** Robotic surgery, Plastic surgery, Head and neck, Lymphatic reconstruction, Microsurgery, Lymphedema

## Abstract

Head and neck lymphedema has gained increasing interest due to improved head and neck cancer survival. However, there exist no guidelines on its management so far. The study reports on the first robotic-assisted lymphovenous anastomosis (LVA) in the head and neck region and further systematically reviews the current literature on lymphatic microsurgery in patients with face and neck lymphedema, aiming to analyze the feasibility and efficacy of different reconstructive techniques. The feasibility of robotic-assisted LVA in the face was assessed and clinical outcomes reported. In addition, a systematic review of the current literature on lymphatic reconstruction of head and neck lymphedema was conducted in accordance with the PRISMA guidelines. The review was registered on PROSPERO (CRD420250651455). Robotic-assisted LVA of the periorbital region led to a significant reduction in edema and improved function. The literature search yielded 468 articles, of which 18 articles were deemed eligible for full-text reading, finally resulting in the inclusion of 10 articles. All included studies showed feasibility of lymphatic reconstruction in the head and neck region and achieved alleviation of lymphedema and its symptoms. However, the small number of patients and heterogenous assessment methods of lymphedema made it difficult to draw definite conclusions on the surgical management of head and neck lymphedema. Robotic-assisted lymphatic reconstruction for face and neck lymphedema is a promising microsurgical technique, however further studies are necessary to create guidelines on the management of head and neck lymphedema.

**Supplementary Information:**

The online version contains supplementary material available at 10.1007/s11701-025-02552-6.

## Introduction

Head and neck lymphedema (HNL) is a burdening condition that manifests as a swelling of soft tissue, originating from the accumulation of lymph fluid due to an impaired function of the lymphatic system. This fluid accumulation leads to chronic inflammation and hence, over time, to tissue fibrosis [1]. The cause of HNL can be primary as a consequence of inherited lymphatic anomalies or secondary, frequently following surgical and/or radiological treatment of head and neck cancer (HNC) [1–3]. An accumulation of lymph fluid can occur both externally (in the soft tissue) and internally (affecting oral cavity, pharynx and larynx), with the latter being relatively underdiagnosed due to its less obvious presentation [3].

Lymphedema in the head and neck region typically leads to a substantial impairment of both physical appearance and function, including restricted eyelid opening and, consequently, vision impairment, as well as difficulties in swallowing, speech, hearing and respiration, resulting in a significantly reduced quality of life (QoL) [4–7].

Previous studies on secondary HNL following HNC treatment reported varying lymphedema prevalences ranging from 12 to 54% [8]. In 2012, a study by Deng et al. even found that 75.3% of 81 study participants presented with either external, internal or combined HNL three months or longer after completion of HNC treatment [9]. This high prevalence was later supported by Ridner et al., who reported a HNL prevalence as high as 90% in HNC survivors [10]. The high variability in prevalence in literature is likely a result of the lack of standardized HNL assessment tools, inconsistent differentiation between internal and external HNL as well as differences in treatment modalities and follow-up time between different study populations [8].

Up to date, various treatment options have been described, including both conservative and surgical approaches. Currently, conservative methods such as complete decongestive therapy (CDT) are considered the gold standard, involving manual lymph drainage, local compression therapy, physical exercises, and skin care [11]. In patients where lymphedema persists despite CDT, reconstructive surgery is considered as an additional treatment option, aiming to restore the lymphatic drainage. In the last two decades, surgical lymphatic reconstruction such as lymphovenous anastomosis (LVA) and vascularized lymph node transfer (VLNT) have gained increasing popularity with a growing body of literature describing its use especially in the treatment of extremity lymphedema [12]. However, more recently several studies reported on surgical lymphatic reconstruction in patients with HNL [13–15]. Apart from the physiological reconstruction of the lymphatic drainage pathway, debulking techniques such as liposuction or tissue excision have been described in HNL treatment—either isolated or in addition to a reconstructive approach [16–18].

The increased attention that HNL has gained may result from the following two reasons: first, improved postoperative survival in patients with HNC, and hence an increased demand for reconstructive surgery, and second the recent discovery of the lymphatic pathways of the brain and its connection to cervical lymphatic structures, with current literature suggesting that the brain’s lymphatic system and its drainage into cervical lymph nodes plays a role in cognitive disability in patients with HNL and in neurodegenerative diseases such as Alzheimer’s disease [1, 19, 20].

In this systematic review we analyze the current literature on surgical treatment of HNL and present, to the best of our knowledge, the first case of robotic-assisted lymphovenous anastomosis in HNL. Hereby, objective and subjective outcomes of HNL following lymphatic reconstruction and surgical aspects are analyzed, aiming to assess the feasibility and efficacy of surgical therapy in HNL, pointing out limitations of current research on this topic and finally discussing the potential of robotic-assisted lymphatic reconstruction in the head and neck region.

## Case report

**Fig. 1:**
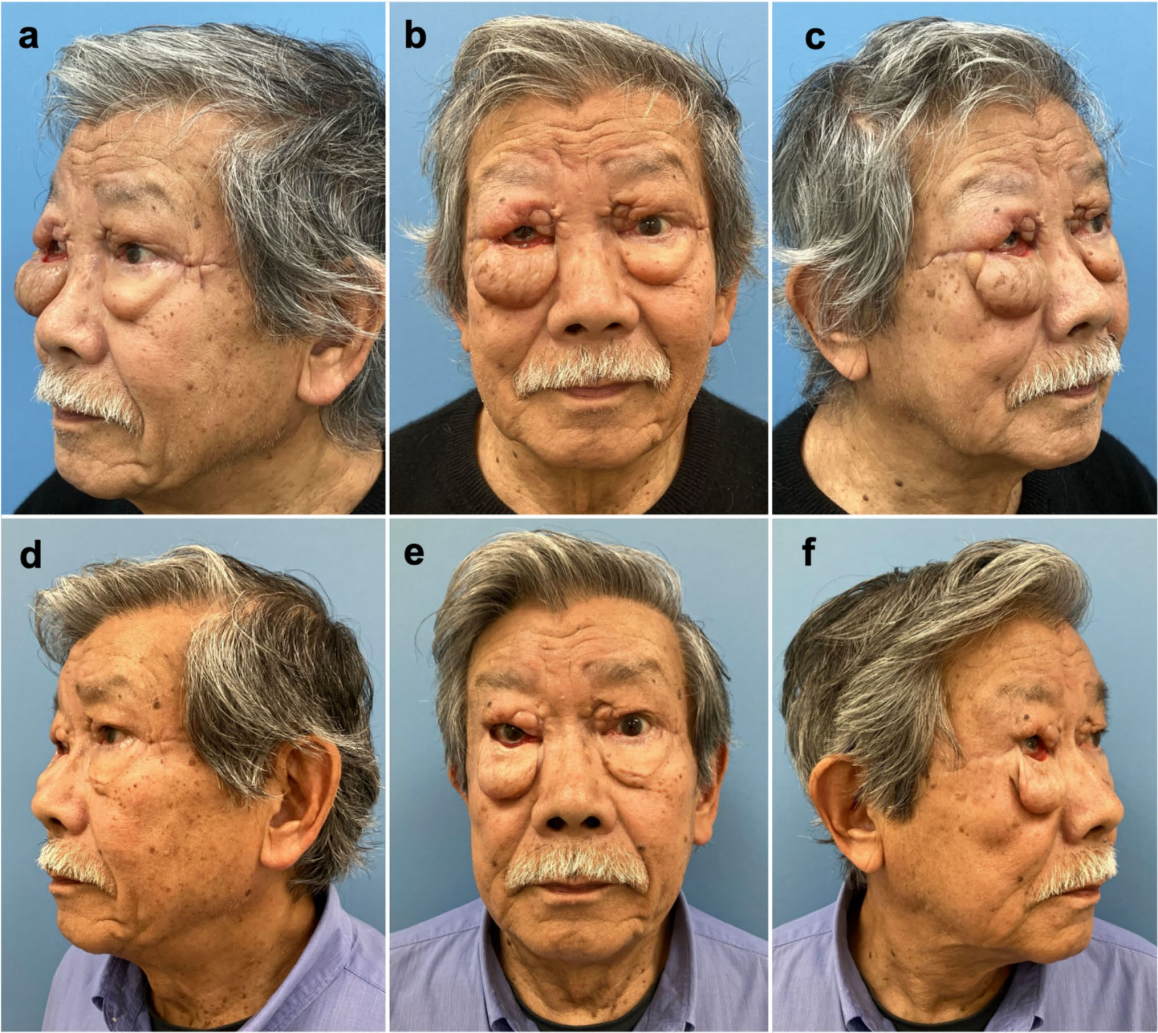
(**a-c**) Preoperative presentation with bilateral periorbital lymphedema, (**d–f)** Three months after robotic-assisted periorbital LVA, eyelid lymphedema and eye closure have substantially improved

A 82-year-old man presented at our outpatient clinic with pronounced bilateral lower and upper eyelid lymphedema. It occurred following upper eyelid blepharoplasty performed six months earlier in an external private practice with postoperative necrotizing infection and a septic shock. The patient’s medical history showed type two diabetes mellitus and arterial hypertension. Upon presentation at our department, the patient had already undergone several debridements due to the extensive bilateral upper and lower eyelid infections (Group A Streptococcus). Subsequently, full thickness skin grafts from the supraclavicular region were performed to reconstruct defects of the upper eyelids on both sides. Almost seven months after the initial event the patient was referred to our outpatient clinic with an impressive edema involving bilateral lower and upper eyelid, which was resistant to conservative lymphatic therapy. The lower eyelids appeared as markedly protruding edematous bags bilaterally, significantly impairing eye closure on the right side (Fig. 1 a-c). The patient specifically reported severe discomfort due to the persistent swelling, impaired visual field, and difficulties with eyelid closure, which substantially impacted his QoL.

**Fig. 2:**
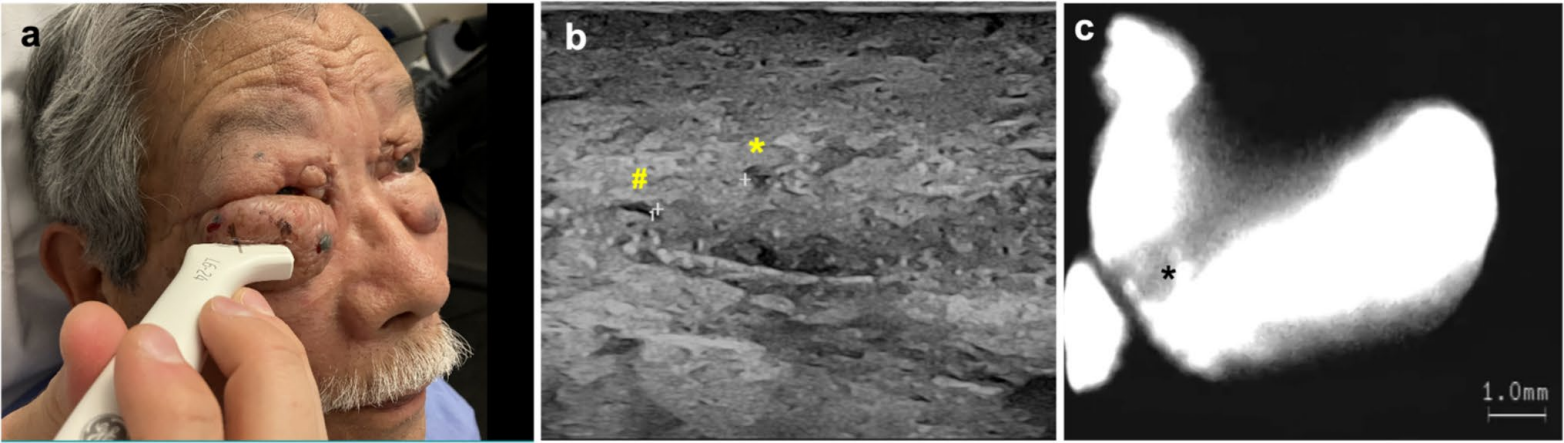
**a** Preoperative high frequency ultrasound (24 MHz) to localize lymphatic vessels and veins in the edematous eyelid tissue, **b** ultrasound image showing a compressible vein (yellow hash) 3 mm next to a lymphatic vessel (yellow asterisk), **c** ICG imaging identifying lymphatic collector

The patient received weekly manual lymphatic drainage without any improvement for four months. To evaluate the lymphatic status, lymphography with intradermal injection of Indocyanine green (ICG) and high frequency ultrasound (24 MHz) was performed (Fig. 2).

**Fig. 3:**
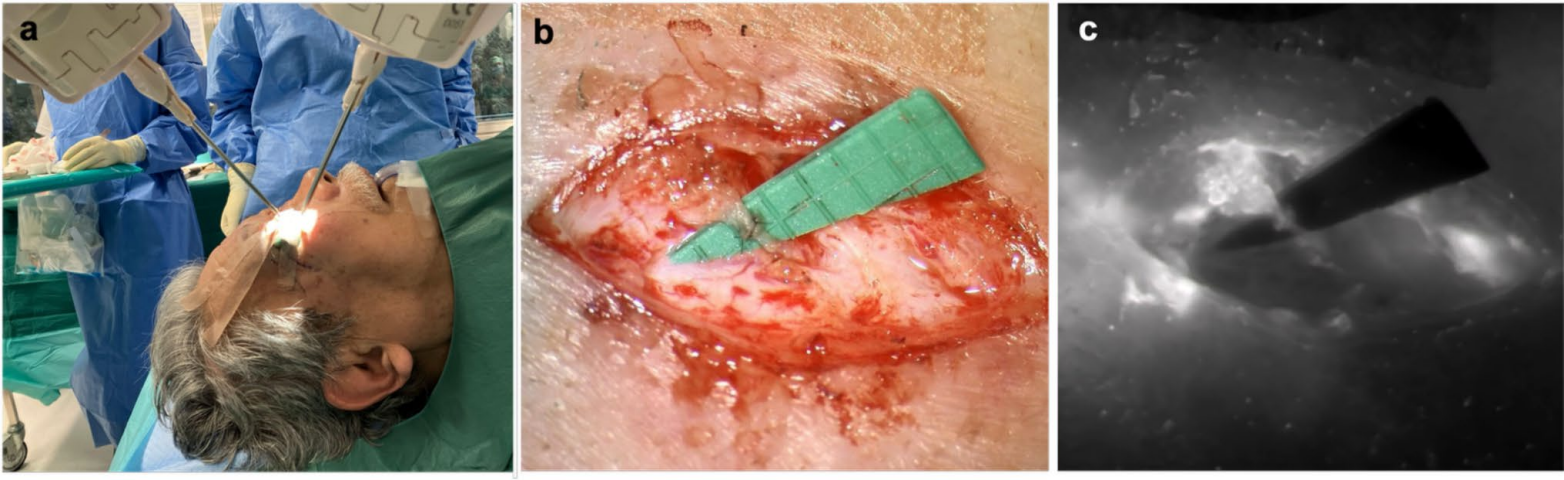
**a** Intraoperative setup of the Symani Surgical System®, **b** A robotic-assisted LVA of a 0.2 mm lymphatic vessel was performed in the lower eyelid on each side of the face, **c** ICG-angiography showing anastomotic patency

Suitable lymph collectors for LVA were identified bilaterally. Robotic-assisted surgery was specifically selected in this intraoperative scenario due to the extremely delicate anatomical structures of the periorbital region and the high precision required for creating reliable LVAs. Intraoperatively, the lymph vessels were illuminated by ICG and a direct approach with an incision at the lower border of the swollen eye bag was performed under general anesthesia. After the dissection, a robotic-assisted LVA with the Symani Surgical System® (Medical Microinstruments [MMI], Jacksonville, FL, USA) using a Nylon 11–0 was conducted on each side (Fig. 3). Further, a canthopexy and minimal tissue debulking were performed on the right eyelid. No postoperative complications occurred.

Twelve days postoperatively, when the patient presented for suture removal, a visible reduction of the edema was already evident bilaterally. The patient resumed manual lymphatic drainage four weeks postoperatively and reported a remarkable reduction of periorbital swelling three months after surgery (Fig. 1 d-f). Furthermore, right eye closure significantly improved and the patient expressed high satisfaction with the results, highlighting a notable improvement in his daily comfort and visual function.

## Systematic review

### Methods

A systematic review of the current literature on surgical treatment of head and neck lymphedema was conducted, adhering to the Preferred Reporting Items for Systematic Reviews and Meta-Analysis (PRISMA) guidelines. For this purpose, the review protocol was registered on PROSPERO, the international prospective register of systematic reviews (CRD420250651455), and a literature search was subsequently performed on March 12, 2025 including the following databases: Medline, Embase, Cochrane CENTRAL, Web of Science Core Collection and Preprint Citation Index, US Clinical trials register, WHO International Clinical Trials Registry Platform, and LILACS. The search was conducted by a medical liaison librarian at University of Zurich using the following and related terms, which were provided by the authors: head and neck lymphedema, lymphatic microsurgery, lymphovenous anastomosis, lymph node transplantation, robotic‐assisted microsurgery. A detailed description of the search strategy can be found in Online Resource 1.

The goal was to explore lymphatic reconstruction of HNL by analyzing objective and subjective outcomes and evaluating different microsurgical techniques. All in-human studies including randomized controlled trials, retrospective studies, case reports and case series were deemed eligible. Conference abstracts, book chapters as well as anatomical and animal studies were excluded from the search. Considering the relative novelty of this topic in medical research, reviews were included in the initial search to avoid excessive narrowing of the search results, and were later sorted and excluded during the screening process. The language was restricted to German and English, and no time restriction was set.

The search results were uploaded to an EndNote (EndNote X9) database and screened by two independent reviewers (CI and CS) based on title and abstract. Subsequently, the same reviewers assessed the included articles for eligibility by full-text screening. Disagreement on inclusion/exclusion was resolved by an additional, independent author (CEZ). A quality assessment of all included studies was performed using the Joanna Briggs Institute (JBI) critical appraisal tools for case reports and case series respectively [21].

The following data of the included studies was extracted and transferred into an Excel file (Microsoft Excel, Version 16, 2024): year of publication, study type, intervention, total number of patients undergoing lymphatic surgery, localization and duration of lymphedema, mean time for anastomosis, anesthesia type, objective and subjective improvement, adverse events, additional pre- and postoperative treatment methods and mean follow-up time. A narrative synthesis with descriptive statistics was performed. Due to the small number of studies included and the large variability of measurement methods, no inferential statistical analysis could be provided.

## Results

**Fig. 4:**
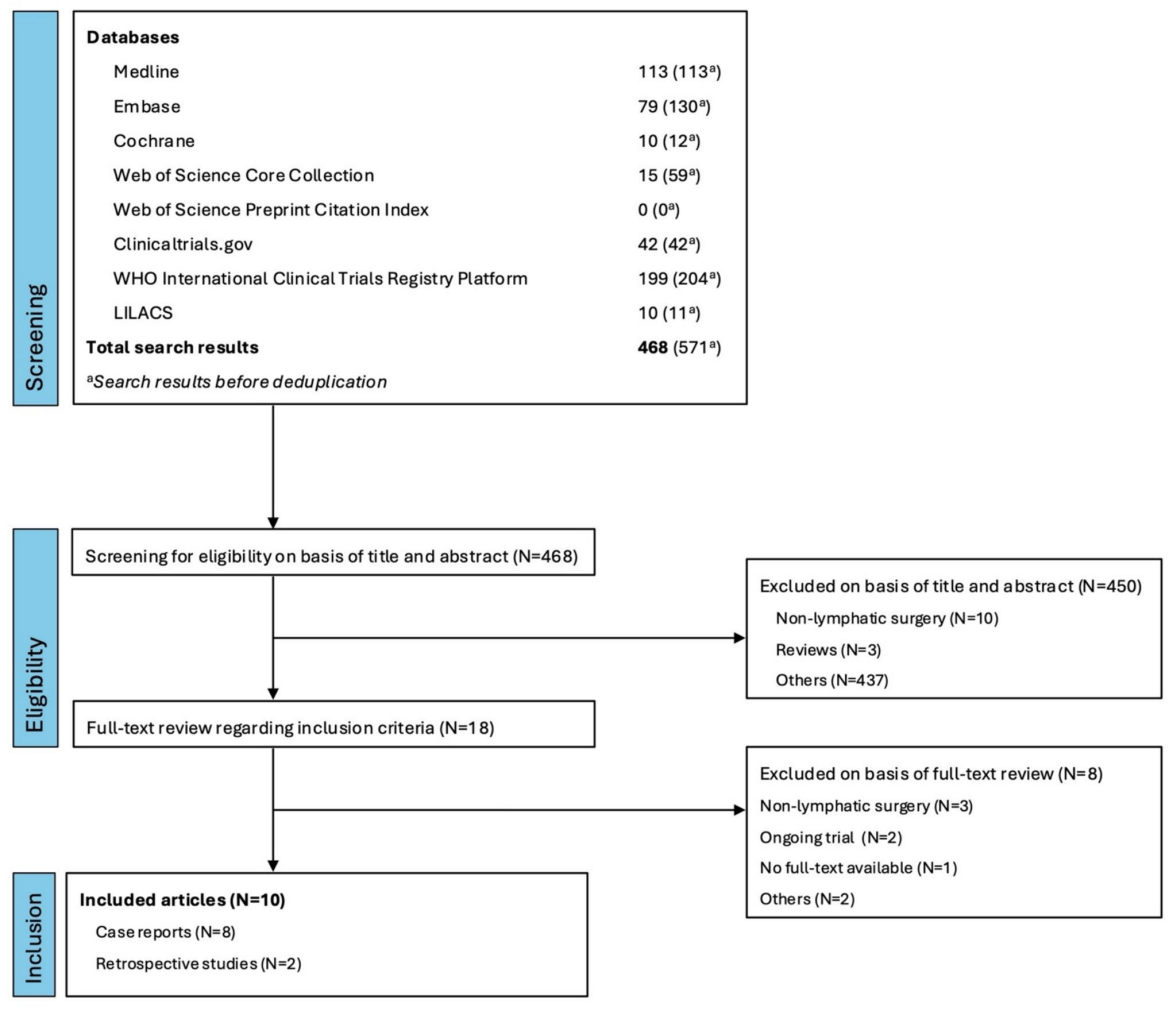
Flowchart of the selection process of articles

The literature search yielded 468 articles after deduplication of the results. The screening based on title and abstract resulted in the inclusion of 18 articles, of which a total of 10 articles were deemed eligible after the full-text review. For one of the 18 articles the full text was neither available online nor was it possible to obtain it from the authors, leading to the exclusion of the article. Furthermore, one study was excluded due to the study population not matching inclusion criteria after being reviewed by the third, independent author following disagreement among the two reviewers. Figure 4 shows the detailed selection process.

### Quality assessment

A quality assessment was conducted using the JBI critical appraisal tool for case reports and case series respectively, addressing the validity and risk of bias for each included study. The assessment of the eight case reports is shown in Online Resource 2. Five out of eight case reports did not meet the criteria of Question 7, which focuses on the reporting of adverse events, thus representing the most frequent risk of bias identified among the included studies [4, 7, 17, 22, 23]. Only one case report lacked fulfillment of two out of eight criteria: first, no transparent description of the diagnostic methods was provided (Question 4) and second, the reported adverse events were unclear (Question 7), allowing no assignment of the different mentioned complications to individual study participants [23]. The two retrospective studies were assessed by the JBI critical appraisal tool for case series shown in Online Resource 3. One retrospective study met all criteria [14], while the preliminary retrospective study provided no clear statement whether there has been a consecutive and complete inclusion of all HNL patients receiving surgical reconstruction during the period of time defined in the study (Question 4 and 5), thus potentially representing a selection bias [24].

### Study Characteristics

The included articles comprise of eight case reports and two retrospective studies, one of which is a preliminary study. Table 1 provides an overview of all studies included.

**Table 1:** Study characteristics and surgical details

Publication	Study type	Intervention (anastomosis, n)	Total patients (n)	Type/Localization of lymphedema	Mean duration of lymphedema (years)	Mean time for anastomosis (min)	Type of anesthesia (local vs general)
Kaciulyte et al. [15] (2025)	Case report	Pretragal LVA	3	Secondary unilateral facial lymphedema	0.92 (range, 0.25–2)	95 ± 6.5 (range, 88–101)	General anesthesia
Cha et al. [17] (2024)	Case report	Periorbital LVA (n = 4); fibrous tissue excision (1.5 × 0.3-cm)	1	Primary unilateral upper eyelid lymphedema	3	NA	General anesthesia
Lin et al. [14] (2024)	Retrospective study	Preauricular (n = 7)/submandibular (n = 1) LVA; preauricular LNVA (n = 2)	6	Secondary internal HNL (n = 3); secondary external HNL (n = 2); secondary combined HNL (n = 1)	0.56 ± 0.36	90–120	NA
Hong et al. [22] (2023)	Case report	Preauricular LVA (n = 2); preauricular LNVA (n = 1)	1	Bilateral periorbital lymphedema (Morbihan disease)	16	NA	Local anesthesia
Hattori et al. [25] (2021)	Case report	Periorbital LVA, debulking blepharoplasty	1	Unilateral periorbital lymphedema (Morbihan disease)	6	NA	Local anesthesia
Inatomi et al. [4] (2018)	Case report	Preauricular LVA	1	Secondary bilateral facial lymphedema	0.25	NA	General anesthesia
Ayestaray et al. [24] (2012)	Preliminary retrospective study	Preauricular π-shaped LVA (n = 5)	4	Secondary HNL	2.6 (range, 1–5)	114 (range, 108–150)	Local anesthesia
Felmerer et al. [23] (2011)	Case report	Lymph vessel transplantation	1	Secondary facial lymphedema	7	NA	NA
Mihara et al. [13] (2011)	Case report	Preauricular and buccal LVA	1	Secondary bilateral eyelid and cheek lymphedema	> 5	NA	Local anesthesia
Withey et al. [7] (2001)	Case report	Tubed deltopectoral flap, “lymphatic bridge”	1	Secondary bilateral HNL	1	NA	NA

*LVA* lymphovenous anastomosis, *LNVA* lymph node-to-vein anastomosis, *HNL* head and neck lymphedema, *NA* not available

A total of 20 patients with HNL receiving different types and combinations of lymphatic reconstruction were included. Surgical reconstructive methods involved LVA, lymph node-to-vein anastomosis (LNVA), lymph vessel transplantation and the usage of a tubed deltopectoral flap as a “lymphatic bridge”. The mean follow-up time was 13.71 ± 11.01 months (range, 0.69–41 months).

All studies reported an improvement of HNL after reconstructive surgery using different methods for quantification of the results, namely facial measurements [15, 24], subcutaneous thickness determination in CT [13] and different surveys and rating scales. The latter encompassed patient-reported outcome measures, namely Lymphedema Symptom Intensity and Distress Surveys—Head and Neck (LSIDS-H&N) and Swallowing Quality of Life (SWAL-QOL) as well as clinician-reported outcome measures, namely MD Anderson Cancer Center Head and Neck Lymphedema (MDACC HNL) rating scale [14]. Six studies used exclusively pre- and postoperative photography as an objective outcome assessment, with five focusing mainly on the reduction of eyelid lymphedema. In addition, in all studies using clinical photodocumentation as assessment tool either the improvement of eyelid function [4, 7, 25], alleviation of symptoms [17, 23] or patient satisfaction [22] was recorded. A detailed description of objective and subjective outcome parameters is shown in Table 2.

**Table 2:** Surgical outcome

Publication	Objective improvement	Subjective improvement	Adverse events	Additional preoperative therapies	Additional postoperative therapies	Mean follow-up time (months)
Kaciulyte et al. [15] (2025)	Lymphedema complete regression (n = 2)/reduction (n = 1) in different facial measurements (e.g. TICd); improved skin softness and pinch test reduction (n = 3); improved eyelid function (n = 3)	Improved skin sensibility (n = 3); reduced discomfort (n = 2)	No adverse events	Conservative decongestive therapy (n = 2)	NA	11 (range, 9–12)
Cha et al. [17] (2024)	Lymphedema regression	Significant symptom improvement	NA	NA	NA	24
Lin et al. [14] (2024)	LSIDS-H&N improved from 1.11 ± 0.54 to 0.44 ± 0.66 (p = 0.02); external lymphedema: MDACC HNL rating scale improved from level 2 to 0 or 1a (p = 0.008); internal: SWAL-QOL improved from 130.5 ± 9.2 to 151 ± 19.8 (p = 0.5)	NA	NA	No additional therapy	NA	15.4 (range, 1–35)
Hong et al. [22] (2023)	Lymphedema reduction	Patient satisfied	NA	Complex lymphatic physiotherapy, micronized flavonoids for 3 months; status post excisional surgery 12 years ago	NA	41
Hattori et al. [25] (2021)	No reexpansion of periorbital region; significant improvement of eye-opening	Improved appearance	No adverse events	Self-applied manual lymph drainage; drug therapy	NA	12
Inatomi et al. [4] (2018)	Lymphedema reduction; full recovery of eye-opening	NA	NA	No additional therapy	No additional therapy	6
Ayestaray et al. [24] (2012)	Improved skin quality (n = 4), average pinch test reduction of 6 mm; significant circumferential reduction (p < 0.02) (n = 3); average circumferential differential reduction rate of 3.7% (range, 0.6–7.8) (p = 0.006); average volume differential reduction rate of 6.9% (range, 2–14.8) (p = 0.05)	Improved skin sensibility (n = 4); improved quality of life (n = 3)	Partial skin necrosis (5 mm) along the tragal scar (n = 1); no postoperative lymphedema worsening	Compression therapy with a nightly elastic stocking; lymph drainage ≥ 6 months	Discontinuation of lymph drainage after 10 months (n = 3)	12
Felmerer et al. [23] (2011)	Distinct lymphedema reduction	Reduction of intermittent pain episodes	NA	Conservative lymph drainage ≥ 6 months	Discontinuation of lymph drainage	3
Mihara et al. [13] (2011)	Lymphedema reduction; subcutaneous tissue thickness reduction of 14 mm in CT; reduced cheek and neck hardness	NA	No adverse events	Self-applied manual lymph drainage for 3 months; compressive bandage	NA	12
Withey et al. [7] (2001)	Lymphedema reduction; improved eyelid-opening	NA	NA	Manual lymph drainage; drug therapy (steroids, antibiotics); bone-conducting hearing aid; tracheostomy and gastrostomy	Manual lymph drainage	0.69

*TICd* tragus-internal canthus distance, *LSIDS-H&N* Lymphedema Symptom Intensity and Distress Surveys—Head and Neck, *MDACC HNL* MD Anderson Cancer Center Head and Neck Lymphedema, *SWAL-QOL* Swallowing Quality of Life, *CT* computed tomography, *NA* not available

### LVA and LNVA

Four studies performed isolated LVAs in the preauricular (patients, n = 9) and the buccal (patients, n = 1) region without any additional surgical intervention [4, 13, 15, 24]. All four studies reported a lymphedema reduction using various measurement methods, namely subcutaneous thickness determination in CT, a combination of different facial measurements (e.g. tragus-internal canthus distance (TICd)), circumferential head measurements and photodocumentation. Ayestaray et al. reported a statistically significant circumferential reduction (p < 0.02) and an improved QoL in three out of four patients following preauricular π-shaped LVA [24]. Inatomi et al. used pre- and postoperative photographs to quantify lymphedema reduction, and additionally the recovery of eye-opening was assessed, showing alleviation of periorbital lymphedema just four days after surgery with the patient being able to open his eyes again [4]. Furthermore, three studies assessed the skin and tissue softness, reporting an improvement in skin quality (n = 2) and sensibility (n = 2) and reduced tissue hardness (n = 1) [13, 15, 24].

Lin et al. treated four patients with isolated preauricular (patients, n = 3) or submandibular LVA (patients, n = 1), one patient with a combination of two preauricular LVAs and a preauricular LNVA, which were performed in three consecutive operations, and one patient with isolated preauricular LNVA [14]. Patients presenting with external type of HNL were assessed with MDACC HNL and LSIDS-H&N, while SWAL-QOL and LSIDS-H&N were used as outcome assessment tool in patients with internal type of HNL. A statistically significant improvement was achieved in LSIDS-H&N (p = 0.02) and MDACC HNL rating scale (*p* = 0.008), while improvement of SWAL-QOL was not significant (p = 0.5).

The combination of preauricular LVA and preauricular LNVA was also successfully used in the treatment of bilateral periorbital lymphedema in a patient with Morbihan disease, described by Hong et al. [22]. In this patient, excisional surgery had already been performed on the lower right eyelid 12 years previously, however the lymphedema recurred. Using microsurgical techniques, namely LVA and LNVA, bilateral lymphedema reduction, which was documented photographically, as well as patient satisfaction could be achieved.

### Combined reconstructive and debulking methods

Two studies described a combination of reconstructive and debulking procedures in periorbital lymphedema. Four periorbital LVAs and subbrow excision of fibrous tissue (1.5 × 0.3 cm) were performed in a patient presenting with primary unilateral upper eyelid lymphedema [17]. The authors described a regression of lymphedema and significant symptom improvement two years postoperatively. The case report by Hattori et al. described a combined approach for treatment of unilateral periorbital lymphedema due to Morbihan disease with one periorbital LVA and concomitant debulking blepharoplasty [25]. One year after the procedure, no recurrent edema of the periorbital region was observed and significant improvement of eye-opening and physical appearance was reported.

### Autologous lymph vessel transplantation

A case report from 2011 by Felmerer et al. reported the performance of an autologous transplantation of lymph vessels from the ventromedial bundle located in the inner aspect of the thigh to the neck region to address secondary facial lymphedema [23]. The lymphatic grafts were proximally anastomosed to preauricular lymphatics of the affected hemiface and distally connected with supraclavicular lymphatics of the contralateral side. During the last reported follow-up after three months, a distinct lymphedema reduction was observed and the patient reported a drastic reduction of intermittent pain episodes. No quantifiable measurements were reported, however photodocumentation was provided.

### Tubed deltopectoral flap as “lymphatic bridge”

In 2001, Withey et al. described a case of severe progressive internal and external facial lymphedema following bilateral neck dissection and radiotherapy, resulting in the impairment of breathing, swallowing, speaking, hearing and eye-opening [7]. Following unsuccessful manual lymphatic drainage and drug therapy, lymphatic drainage was re-established by introducing a tubed deltopectoral flap into the dermis of the cheek acting as a “lymphatic bridge” between the patient’s trunk and his face thus bypassing the site of obstruction. Three weeks after surgery, facial lymphedema decreased and the patient was able to open his left eye again. The study provided no further information on the course of symptoms or subsequent follow-ups.

## Discussion

### Case report

To the best of our knowledge, we herein report on the first robotic-assisted lymphovenous anastomosis in the face on a 0.2 mm lymphatic vessel. Reduction of bilateral upper and lower eyelid lymphedema was achieved by bilateral periorbital LVA with additional tissue excision on the right lower eyelid and canthopexy on the right eye.

In accordance with the literature review provided in this study, our case report confirmed the feasibility and efficacy of LVA in the management of HNL and further demonstrated the successful usage of robotic assistance in HNL. Thereby, an expansion of the previous application of microsurgical robotic platforms in the treatment of extremity lymphedema and central lymphatic lesions, which has gained a growing body of literature over the past decade, was achieved [26, 27]. The Symani Surgical System® could further improve the outcome of surgical HNL treatment by providing the microsurgeon with enhanced precision, increased range of motion through wristed instruments, improved ergonomics and facilitated access to narrow anatomical sites such as the oral cavity [28, 29]. Recently, a consensus among 13 European microsurgery centers using microsurgical robotic platforms was published, defining higher precision as the top benefit of robotics and further, lymphatic surgery as the top indication for the usage of robotic assistance in microsurgery [30]. In our patient, the use of a microsurgical robotic platform enabled the performance of LVAs on delicate lymphatic vessels measuring 0.2 mm in diameter. Additionally, the freestanding robotic system eliminated the need to place the surgeon’s hands on the head of the patient, thus preventing the surgical field from moving when relocating the hands, and further ensuring an ergonomic positioning of the surgeon.

### Surgical techniques and outcomes

The systematic review provides a comprehensive overview of different microsurgical reconstructive techniques used in patients with HNL. All 10 included articles reported successful reduction of HNL after surgical lymphatic reconstruction, confirming the feasibility and the potential of lymphatic surgery in the treatment of HNL.

With eight out of 10 included studies using LVA for restoration of lymphatic drainage, it represents the most commonly used lymphatic reconstructive technique in the head and neck region. The use of LVAs has already been widely established as a treatment method for extremity lymphedema, especially in early stages of the disease, where little fibrosis is present [31].

Another widely used surgical approach in extremity lymphedema is VLNT [32], however, to date, we found no report of the use of this technique in the treatment of HNL. A possible reason for this is that a VLNT adds volume to the recipient site, which may not be favorable for an aesthetically pleasing outcome in the head and neck region. Furthermore, VLNT is more invasive than LVAs involving a donor site and leading to longer operating times.

Several authors reported lymphedema recurrence following isolated performance of debulking techniques, hence a combination of LVA and tissue debulking may be a viable option to improve long-term outcomes of eyelid lymphedema [33–35]. Using a combined approach, two case reports achieved regression of eyelid lymphedema without recurrence during a 1- and 2-year follow-up [17, 25]. In our case, we chose a combined approach of LVA and tissue excision only on one side of the face. The potential benefit of a combined approach should be further investigated by direct comparison with the sole performance of isolated LVA and isolated debulking surgery.

An advantage of LVA is its minimal invasive nature, thus allowing to operate under local anesthesia, which was reported in four case reports [13, 22, 24, 25]. Two case reports using local anesthesia performed either LNVA or blepharoplasty in addition to LVA [22, 25].

Lymph vessel transplantation was only used in one case by Felmerer et al. and solely evaluated by visual assessment three month post-surgery, making it difficult to determine effectiveness of the technique in HNL [23]. However, the reduction of intermittent pain episodes and the discontinuation of lymph drainage reported in the study indicate a positive outcome. The authors however pointed out the technical difficulty of raising 25–30-cm-long lymphatic vessels, suggesting that this prevents widespread use of this method. Another downside of lymph vessel transplantation is the need for a donor site, making it more invasive than LVA.

More than two decades ago, Withey et al. achieved substantial lymphedema reduction using a tubed deltopectoral flap to bypass obstructed cervical lymphatics. A surgical approach was chosen after futile attempts of conservative treatment, achieving substantial alleviation of symptoms within a few weeks. Even though a modern approach would most likely prefer a less invasive reconstructive technique such as LVA, this case underlines the value of surgical restoration of lymphatic drainage in patients with severe facial lymphedema.

Mihara et al. pointed out the potential risk of promoting metastasis formation by restoring lymphatic drainage in patients with previously treated HNC [13]. Hence, the performance of a careful follow-up for exclusion of cancer recurrence is crucial prior to lymphatic reconstruction. Hirche et al. suggest a successful completion of cancer therapy and a period of 6 to 12 months without tumor recurrence as a prerequisite for surgical lymphatic reconstruction to avoid the unintended promotion of tumor spreading [36]. Seven of the included studies described lymphatic reconstruction in patients with secondary HNL following HNC therapy, with the mean duration between diagnosis of lymphedema and lymphatic reconstruction amounting to 2.48 ± 2.58 years (range, 0.25–7 years) [4, 7, 13–15, 23, 24]. Inatomi et al. performed a LVA for palliative purposes in a patient presenting with a metachronous skin metastasis of a maxillary sinus carcinoma, which had been surgically treated three months earlier, thus accepting the risk of further metastasis spreading for the improvement of QoL [4].

### Limitation and future prospects

This systematic review aimed to critically analyze the existing literature on surgical lymphatic reconstruction in HNL and draw conclusions for future management of patients with HNL. All 10 included articles showed, that reconstructive surgery of HNL is safe and feasible. However, there are different limitations of the studies and consequently of this review, which make it difficult to provide concise recommendations in regards to the effectiveness and superiority of individual techniques. Firstly, all studies used a retrospective study design, with eight studies being case reports, consisting of small patient cohorts. However, all studies showed low risk of bias using the JBI critical appraisal tool for case reports and case series respectively. Further, only two studies conducted statistical testing of their results. The heterogenous assessment of HNL, and the fact that six studies lacked of the quantification of lymphedema altogether, made it difficult to compare the surgical outcomes of different studies and limited this review to the conduction of a narrative synthesis with descriptive statistics.

With future research providing randomized controlled trials with larger patient cohorts, the benefit of surgical reconstruction in comparison to non-surgical treatment can be evaluated to develop guidelines for HNL therapy. Current assessment tools of HNL include patient-reported outcome measures such as LSIDS-H&N and SWAL-QOL [37, 38], clinician-reported outcome measures such as MDACC HNL rating scale [39] and other facial measurement methods taking point-to-point or circumference measurements as described in two studies included [15, 24], as well as imaging-based assessment methods such as CT or ultrasound [40, 41]. However, further studies are needed to evaluate reliability of these measurement methods and create a gold-standard for HNL assessment [42].

## Conclusion

This systematic review supports previous findings, that microsurgical lymphatic reconstruction is a safe and feasible treatment option for HNL, with LVA in particular demonstrating promising results regarding lymphedema reduction and improvement in skin quality across several studies. However, the current literature is limited by significant heterogeneity, small patient cohorts, and inconsistent outcome measures, which restrict direct comparisons across studies. In this context, we report the first robotic-assisted LVA in the head and neck region, demonstrating both feasibility and advantages of robotic assistance, particularly regarding higher precision and improved ergonomics. Nonetheless, the robotic-assisted technique presents limitations, including increased operative costs, extended setup times, and the requirement for specific surgical training. Further prospective studies are essential to robustly assess the value and cost-effectiveness of robotic assisted approaches compared to conventional microsurgical techniques. Additionally, introducing a universal assessment tool for HNL would significantly enhance the comparability of future studies and treatment modalities.

## Supplementary Information

Below is the link to the electronic supplementary material.

Supplementary file1 (XLSX 51 KB)

Supplementary file2 (XLSX 10 KB)

Supplementary file3 (XLSX 10 KB)

## Data Availability

No datasets were generated or analysed during the current study.
